# Repurposing Combination Therapy of Voacamine With Vincristine for Downregulation of Hypoxia-Inducible Factor-1α/Fatty Acid Synthase Co-axis and Prolyl Hydroxylase-2 Activation in ER+ Mammary Neoplasia

**DOI:** 10.3389/fcell.2021.736910

**Published:** 2021-11-18

**Authors:** Lakhveer Singh, Subhadeep Roy, Anurag Kumar, Shubham Rastogi, Dinesh Kumar, Mohd. Nazam Ansari, Abdulaziz S. Saeedan, Manjari Singh, Gaurav Kaithwas

**Affiliations:** ^1^Department of Pharmaceutical Sciences, Babasaheb Bhimrao Ambedkar University, Lucknow, India; ^2^Center for Biomedical Research, Sanjay Gandhi Post Graduate Institute of Medical Sciences, Lucknow, India; ^3^Department of Pharmacology and Toxicology, College of Pharmacy, Prince Sattam Bin Abdulaziz University, Al-Kharj, Saudi Arabia; ^4^Department of Pharmaceutical Sciences, Assam University, Silchar, India

**Keywords:** mammary gland carcinoma, hypoxia inducible factor-1α (HIF-1α), fatty acid synthase (FASN), prolyl hydroxylase-2, voacamine, repurposable drugs

## Abstract

The current study investigated the role of combination therapy with voacamine and vincristine in preventing mammary gland carcinoma through prolyl hydroxylase-2 activation. Prolyl hydroxylase-2 activation leads to the downregulation of hypoxia-inducible factor-1α and fatty acid synthase. Overexpression of hypoxia-inducible factor-1α and fatty acid synthase has been previously reported in solid tumors of the mammary gland. After screening a battery of natural compounds similar to vincristine, voacamine was selected as a possible prolyl hydroxylase-2 activator, and its activity was evaluated using a 7,12-dimethylbenz[a]anthracene-induced rat model. The combination therapy was evaluated for cardiac toxicity using a hemodynamic profile. Angiogenic markers were evaluated by carmine staining. Monotherapy and combination therapy were also evaluated for liver and kidney toxicity using hematoxylin and eosin staining. The antioxidant potential was delineated using oxidative stress markers. The serum metabolomic profile was studied using NMR spectroscopy, and the disruption of fatty acids was evaluated using gas chromatography. Western blotting of proteins involved in hypoxic pathways was performed to decipher the action of therapy at the molecular level. Immunoblotting analysis validated that combination therapy has potential toss with prolyl hydroxylase-2 activity and thus initiates proteolytic degradation of hypoxia-inducible factor-1α and its consequent effects. Combination therapy also stimulated programmed cell death (apoptosis) in rapidly dividing cancer cells. The present study explored the role of voacamine inactivation of prolyl hydroxylase-2, which can decrease the overexpression of hypoxia-inducible factor-1α and fatty acid synthase in mammary gland carcinoma cells.

## Graphical Abstract

Mechanism of VOA and VIN to inhibit fatty acid synthesis in DMBA-induced mammary gland carcinoma of albino Wistar rats. Hypoxia-activated HIF-1α enhances lactate acidosis in the tumor microenvironment, and dysregulated pH in the tumor microenvironment activates SREBP-1c and FASN expression to speed up the fatty acid synthesis required for plasma membrane synthesis in rapidly proliferating cells. VOA- and VIN-activated PHD-2 enhanced the proteolytic degradation of HIF, thus inhibiting fatty acid synthesis. HIF-1α, hypoxia-inducible factor-1α; SREBP-1c, sterol regulatory element-binding protein-1c; FASN, fatty acid synthesis; PHD-2, prolyl hydroxylase-2.

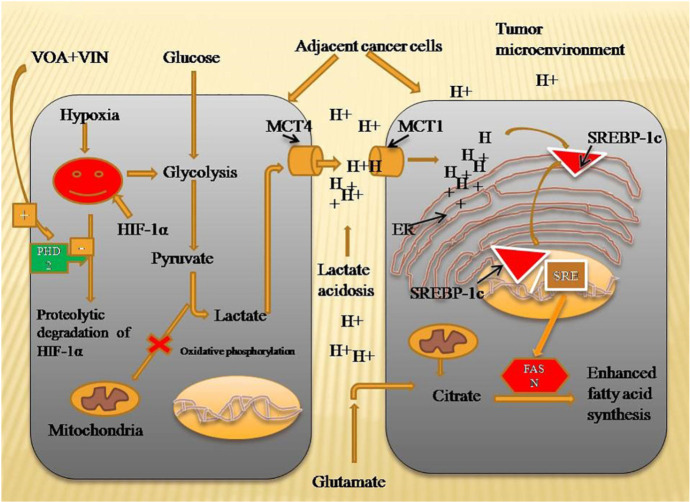

## Introduction

Mammary gland carcinoma is the most commonly observed malignancy in women. It has been reported that mammary gland cancer makes up [as per the World Health Organization [WHO], 2020 report, 2.3 million women were diagnosed with cancer and 685,000 die of it^1^ ] one-fourth of all cancers diagnosed every year worldwide (Zhang et al., 2012). When mammary gland growth and proliferation of mammary gland tissue are not under control, they divide and form a lump of cells that transform into cancer if they remain undiagnosed (Ciriello et al., 2016). Despite much progress in the field of cancer radiation and chemotherapy, the treatment and management of mammary gland carcinoma are still very difficult (Marcotte et al., 2016). Moreover, failure of radiation and chemotherapy due to hypoxia in solid tumors of the mammary gland is another encountered problem (Guo et al., 2018). Many studies have revealed that hypoxia-inducible factor-1α (HIF-1α) is the main component that makes various decisions to attain a more hostile environment for proper growth and development of tumor cells even in the absence of oxygen (Singh et al., 2016). It enhances the expression of various genes involved in the glucose and fatty acid synthesis pathway (Singh L. et al., 2018). To synthesize their plasma membrane, rapidly dividing cancer cells require fatty acids, especially polyunsaturated fatty acids (PUFAs), in much larger quantities than normal dividing cells (Roy et al., 2020). Therefore, cancer cells adopt an alternative pathway to meet their fatty acid requirements. Here, the mammary gland cancer tissues utilize more glucose and glutamate for their production. Thus, from the above discussion, it is clear that fatty acids play a vital role in the growth and development of mammary gland cancer cells because of the overexpression of fatty acid synthase (FASN).

Prolyl hydroxylase-2 (PHD-2) is an essential regulator of HIF-1α and executes hydroxylation and the degradation of HIF-1α in the presence of oxygen and maintains it to a minimum level in normal cells (Ratcliffe et al., 2017). However, in a solid hypoxic tumor, reduced levels of oxygen deactivate PHD-2 and activate HIF-1α. Previously, our research group hypothesized that the chemical activation of PHD-2 could curtail the increased levels of HIF-1α and FASN in mammary gland carcinoma (Singh M. et al., 2018; Devi et al., 2019).

Instead of discovering and developing a new chemical compound, repurposing already known drugs could be a better and time-saving strategy against various disorders (Sharma et al., 2020). More recently, working in the same line, our research group demonstrated the role of activation of PHD-2 by voacamine (VOA) alone and in combination with tamoxifen (TMX) in the treatment of ER+ mammary gland carcinoma induced by *N*-methyl-*N*-nitrosourea (MNU) in albino Wistar rats. Metabolomics and western blotting results revealed the activation of PHD-2 and downregulation of HIF-1α, sterol regulatory element-binding protein-1c (SREBP-1c), and FASN with a low VOA dose (1 mg/kg, s.c.) and with its combination with TMX (1 mg/kg, p.o.). The study also demonstrated the toxicity of high doses of VOA (2 mg/kg, s.c.) in the experimental animals, as toxic group-like manifestations were observed after treatment. A reduction in alveolar buds (ABs) and branching has also been reported to have an antiangiogenic effect on the drug (Singh et al., 2021). Since VOA has shown promising results in restoring proteins of the hypoxic pathway and serum metabolites of MNU-induced mammary gland carcinoma in experimental animals, we designed another study to validate our findings.

The purpose of repurposing vincristine (VIN) with VOA was to fulfill the following three objectives: First, we used another carcinogen, 7,12-dimethylbenz[a]anthracene (DMBA), to validate the above findings. DMBA is an even more powerful carcinogen than MNU, so experimental animals will develop more extensive visible tumors with significant hypoxia. DMBA is a polycyclic aromatic hydrocarbon that induces mammary gland tumors by its epigenetic mechanism on DNA. After reaching the cytosol of mammary gland cells, it binds with the aryl hydrocarbon receptor (AhR), which translocates to the nucleus and associates with the cofactor AhR nuclear translocator (ARNT). The AhR/ARNT adduct binds on the specific regions of the DNA and induces gene transcription. The initial tumorigenesis effect of DMBA involves activation of cytochrome 450, which metabolizes DMBA into mutagenic epoxide intermediates that form DNA adducts. These adduct binds with the DNA mutations and induces carcinogenesis in mammary cells. MNU, on the other hand, initiates its carcinogenesis effect by the alkylation of guanine residue of DNA on purine. Second, as the results of our MNU study confirmed that stabilization of HIF-1α in hypoxia increases the expression of FASN (responsible for *de novo* fatty acid synthesis), we could not determine the fatty acid profile of experimental animals. Therefore, in the DMBA study, we analyzed the fatty acid profiles of control and toxic control animals using mammary gland homogenates by gas chromatography (GC). Third, we determined the effect of VOA and its combinatorial drug on the mitochondrial apoptotic pathway, which we could not determine in the MNU study.

Fourth, we will determine the safety of VOA monotherapy and its adjuvant therapy in vital organs such as the kidney and pancreas by histopathology of liver and kidney tissue. Last but not least, this time, we will start our therapy with VOA alone and its combination with VIN. Why did we choose to combine VOA with VIN while we used TMX with VOA in our initial study? This is because TMX requires estrogen receptors on the target cells and hence can stop the growth and proliferation of ER+ mammary gland carcinoma cells only. Therefore, combining VOA with TMX in the MNU study proved that VOA works well in ER+ mammary gland neoplasms. However, its effectiveness in triple-negative breast carcinoma (TNBC) is doubtful and remains undetermined. This is one reason why TMX with VOA was not used. The second question is, what is the rationale for using VOA with VIN? We chose VIN with VOA for two reasons. First, VIN is an effective drug for the treatment of TNBC. VIN is a well-known anticancer drug that acts by inhibiting microtubule formation in target cells. To initiate its chemotherapeutic effect, VIN must reach and accumulate in the target tissue. However, it fails to do so because, under hypoxia, cancer cells express highly patterned vasculogenic mimicry (VM) channels and RLIP76 anti-apoptotic protein, thereby imparting resistance to the tumor cells (Benito et al., 2011; Zeng et al., 2015). Here, VOA has already been proven to work in hypoxia, and its combination with VIN could enhance its efficacy.

In the present study, we aimed to demonstrate the role of the natural compound VOA alone and in combination with VIN in mammary gland chemoprevention through activation of PHD-2 and subsequent downregulation of HIF-1α and FASN.

## Materials and Methods

### *In silico* Study

**FIGURE 1:**
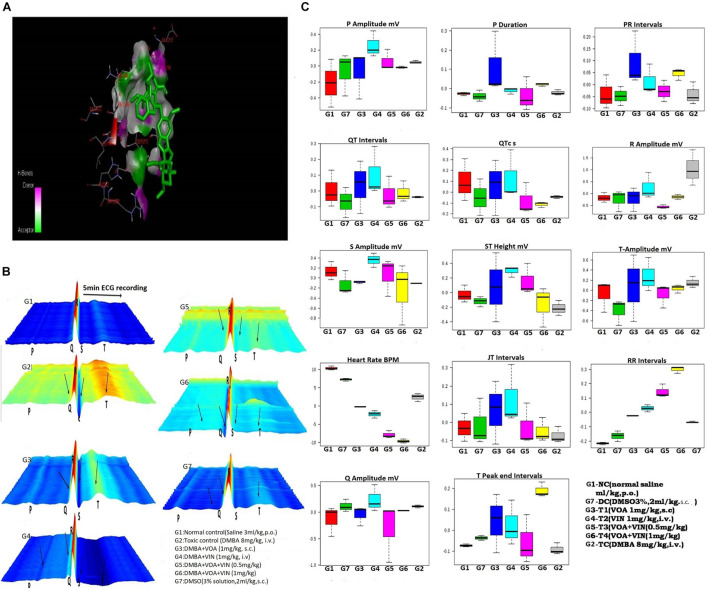
**(A)** Docked pose of VOA with the PHD-2 protein. Docking of VOA with PHD-2 was performed using the Auto Dock Tools 4.2. VOA formed hydrogen bonds with LEU193, LEU191, HIS282, ARG282, ARG281, ALA190, LEU188, PHE213, GLY213, LEU214, GLU217, ASP278, SER275, SER214, LEU271, and THR218, with a binding energy of –7. **(B)** Waterfall map presentation of the ECG/HRV recording of experimental animals. In the figure, the deformities in the ECG are indicated by arrows. Groups are as follows: NC, normal control (saline 2 ml/kg); G2, toxic (DMBA 8 mg/kg, i.v.); G3, VOA (1 mg/kg, s.c.); G4, VIN (1 mg/kg, i.v.); G5, VOA (0.5 mg/kg) + VIN (0.5 g/kg, i.v.); G6, VOA (0.5 mg/kg) + VIN (0.5 g/kg, i.v.); and G7, DMSO (3%, s.c.). **(C)** Box-cum-whisker plot of the ECG/HRV recording of experimental animals. 1 (red): NC (normal saline 2 ml/kg, p.o.); 2 (green): DC (DMSO 3%, 2 ml/kg, p.o.); 3 (blue): G3 (VOA 1 mg/kg, s.c.); 4 (light green): G4 (VIN 1 mg/kg, i.v.); 5 (pink): G5 (VOA+VIN 0.5 mg/kg); 6: G6 (1 mg/kg); 7: G2.

*In silico*, drug screening methods are currently in trend to reduce the number of animals used in drug development. Sophisticated docking software like AutoDock is freely available online and can be used to predict the energetically favorable binding sites present on the particular receptors (Gupta et al., 2018). To carry out an *in silico* study, a library of natural compounds was created using the Zinc database. The compounds were structurally similar to VIN. All the compounds were docked (AutoDock 4.2) with the PHD-2 protein [Protein Data Bank (PDB) ID: 2NC9], and approximately 13 compounds were found to have good binding energy with the PHD-2 protein. After all the compounds were analyzed, VOA (Zinc ID 169368472) was selected for the *in vivo* study (Figure 1A).

### *In vivo* Study

#### Chemicals and Reagents

Cytocristin (Cipla) (VIN) was purchased from a local market. VOA (NCI-3375-85-5) was obtained from the National Cancer Institute (NCI) in the United States as a drug sample. Dimethyl sulfoxide (DMSO) (Merck, D8418), Ponceau S (HiMedia, ML045), hematoxylin (HiMedia, So58), eosin (HiMedia, S007), acrylamide (Genetix, 1443c196), transfer buffer (Genetix, GX-9411AR), glycine (Amresco, 0167-kg), ammonium persulfate (APS) (Loba Chemie, LB2282a09), radioimmunoprecipitation assay (RIPA) lysis buffer (Amresco, N653), tetramethylene diamine (TEMED) (Amresco, 0761), Tris UltraPure (Duchefa Biochemie), sodium lauryl sulfate (SLS) (Loba Chemie, S56971301), DMBA (Sigma-Aldrich, 57-97-6), protein assay kit (Amresco, M173), bovine serum albumin (BSA) (Genetix, PG-2330) were also obtained. All other chemicals used in this study were purchased from Genetix Asia Pvt., Ltd., New Delhi, India, and all were of molecular grade.

#### Experimental Animals

Female albino Wistar rats were obtained from the Central Drug Research Institute (CDRI), Lucknow, after the approval of the protocol (UIP/IAEC/May-2016/06). After procurement, the animals were kept in a central animal house in the Department of Pharmaceutical Sciences (BBAU). The animals were kept under a 12-h light/dark cycle at a stable temperature of 22–24°C and humidity. In addition, the animals had free access to standard animal feed water *ad libitum*. All experiments were performed according to the guidelines laid by the Committee for Control and Supervision of Experiments on Animals (CPCSEA), Government of India. After 1 week, animals were randomly selected (in the weight range 100–150 g) and divided into seven groups containing eight animals each: Group 1 [normal control (NC) receives normal saline 3 ml/kg, p.o.]; Group 2 (toxic control receives DMBA 8 mg/kg, i.v.); Group 3 (G3) receives DMBA 8 mg/kg, i.v. + VOA 1 mg/kg, s.c.); Group 4 (G4) receives DMBA 8 mg/kg, i.v. + VIN 1 mg/kg, i.v.); Group 5 (G5) receives DMBA 8 mg/kg, i.v. + VOA 0.5 mg/kg, s.c. + VIN 0.5 mg/kg, i.v.); Group 6 (G6) receives DMBA 8 mg/kg, i.v. + VOA 1 mg/kg, s.c. + VIN 1 mg/kg, i.v.); Group 7 (dummy control receives 3% DMSO solution s.c.) (Supplementary Table 1). DMBA was prepared in a 3% DMSO solution and administered once at the beginning of the study. Normal saline, 3% DMSO solution, and drug treatment were administered after 15 days of DMBA injection and continued for up to 1 month at 1-week interval. The study was continued for up to 3 months. At the end of the study, blood was collected from the retro-orbital plexus to study the metabolomic profiles of the control and treatment groups. After blood withdrawal, the animals were sacrificed under mild ether anesthesia by cervical dislocation. The abdominal cavity was opened through a median incision. The mammary gland was carefully separated from the skin using sharp scissors and forceps and mounted on a slide to carry out carmine staining. The liver and kidney were also separated and preserved at −20°C to assess the toxicity of drug molecules.

#### Hemodynamic Analysis

The hemodynamic profile was measured using an AD instrument to assess the cardiac toxicity due to DMBA. After anesthetization, the animals were mounted on wax trays by injecting ketamine hydrochloride (50 mg/kg, i.m.) and diazepam (2.5 mg/kg, i.m.). The dorsal and ventral thorax skin was cleaned and sterilized with spirit, and then platinum hook electrodes were fixed on it to record the electrocardiogram (ECG) signals. These electrodes were connected to a bio-amplifier (ML-136) and a channel power lab (ML-136). Both instruments work together to convert analog signals into digital signals (AD Instruments, Australia) stored on the system’s hard disk. Offline analysis of the saved ECG signals was performed using Lab Chart Pro-8 (AD Instruments, Australia) (Rawat et al., 2019).

Electrocardiogram (ECG) signals were also employed for the heart rate variability (HRV) analysis. First, a manual inspection of the recorded signals was performed to detect R waves accurately. Subsequently, the R waves per unit time were plotted to calculate the heart rate (HR). Then, the HR was calculated by plotting the number of R waves per unit time. Finally, Lab Chart Pro-8 (AD Instruments) was employed to calculate the time and frequency domain parameters of HRV (Mishra et al., 2016; Sammi et al., 2018).

#### Carmine Staining

For whole-mount analyses of mammary glands, mammary gland tissues were removed from the rats and stretched over the glass slides. After drying, the slides were placed in Carnoy’s fixative solution [ethanol (60%), chloroform (30%), and glacial acetic acid (10%)] for 2 h and then washed with decreasing concentrations of ethanol (90, 70, 35, and 15%) for 1 h. The slides were placed in the alum carmine stain (1 g carmine dye and 2.5 g aluminum potassium sulfate in 500 ml distilled water). After 2 days, the carmine-stained slide was removed and dehydrated with an increasing concentration of ethanol (70, 95, and 100%), and the lipids were removed by overnight immersion in xylene. Slides were examined under a biological microscope at × 4 in order to check the presence/absence of ABs, terminal end duct (TED), terminal end bud (TEB), and lobules (LOs) (Manral et al., 2016; Roy et al., 2019).

#### Histopathological Analysis

Hematoxylin and eosin (H&E) staining was performed to examine the mammary glands, liver, and kidneys. First, tissues were placed in a 10% formaldehyde fixing solution and then buried inside wax cubes. With the microtome, 5-μm sections were prepared and stained with H&E. Finally, the prepared tissue sections were examined under a digital biological microscope (BR Biochem Life Sciences, N120, New Delhi, India) to visualize and image the stained tissue. Photographs were taken at × 4 and × 40 (Gautam et al., 2018b).

#### Antioxidant Markers

Frozen mammary gland tissue samples were thawed, precisely weighed, and homogenized in 0.15 M KCl. The mixture was centrifuged at 10,000 × *g* for 15 min. The supernatants were collected and kept in an ice bath until analysis. Enzymatic assays for catalase, thiobarbituric acid reactive substances (TBARS), superoxide dismutase (SOD), protein carbonyl (PC), and glutathione (GSH) were determined according to a previously described method (Yadav et al., 2017; Singh et al., 2019). The reactivity of the enzymes with tissue samples was determined spectrophotometrically using an ultraviolet (UV)–visible spectrophotometer (Agilent Technologies, Cary 60).

#### Fatty Acid Methyl Ester Analysis

Frozen mammary gland tissue was accurately weighed and dissolved in a mixture of chloroform and methanol (2:1) to obtain a 0.5% tissue homogenate. The homogenized tissue was further sonicated at 4°C for up to 5 min and then filtered using Whatman filter paper. Methanol was added to make up the final volume. In the filtrate, 0.2 ml double-distilled water was added to remove the nonfatty components. The resultant mixture was left undisturbed for a minute. After a minute, the solution was centrifuged at 5,000 × *g* for 5 min. After centrifugation, the lower lipid-containing layer was conserved, and the upper nonfatty layer was discarded. In the subsequent step, methyl esters of the lipid sample were prepared by stirring the sample (0.75 g) with hexane (2 ml) and 0.2 ml methanolic KOH (2N). The resultant mixture was vortexed for 15 min to separate it into two layers. Fatty acid methyl ester (FAME) is present in the upper layer. It was carefully separated and used to analyze the control, toxic, and treatment groups (Gautam et al., 2018a; Roy et al., 2018).

#### ^1^H-NMR Study

^1^H-NMR was performed on the blood serum samples. Serum samples were thawed and centrifuged. For data acquisition, 220 μl of the supernatant was collected in NMR tubes (Wilmad LabGlass, United States). Two hundred twenty microliters of (NMR) buffer solution (20 mm sodium phosphate saline, pH 7.4, prepared in D_2_O) was added. After this, a 2-mm sealed tube called co-axial containing a 0.5 solution of 3-trimethylsilyl-(2,2,3,3-d4)-propionic acid (TSP) was inverted in 5-mm NMR tubes, and 150 μl of the solution was poured into it. It worked as an internal reference standard. The prepared samples were analyzed using an NMR spectrometer (Bruker NMR spectrometer) at 800 MHz, and the raw spectra were obtained as NMR peaks. Additionally, Carr–Purcell–Meiboom–Gill (CPMG) NMR spectra were recorded for each serum sample by adopting the Bruker standard pulse program library sequence (cpmgpr1d) with the saturation of the water peaks. The spectra were further processed using the Bruker software TopSpin v2.1 (Bruker BioSpin GmbH, Silberstreifen, 476287 Rheinstetten, Germany) and AMIX software to perform spectral binning of the CPMG data. The binned data were then further submitted to MetaboAnalyst to carry out multivariate analysis of the metabolomic spectral data. First, principal component analysis (PCA) was performed to obtain an initial overview of the metabolites in the control, toxic, and treatment groups. Next, data were again analyzed using the partial least squares discriminant analysis (PLS-DA) method to identify the metabolites responsible for the class separation among the grouped animals. The data were Pareto-scaled for both PCA and PLS-DA and strictly validated for statistical significance. The R2 and Q2 parameters described the cross-validation of the models, and *p*-values ≤ 0.05, calculated with the Mann–Whitney test for pairwise comparisons, were assumed to be statistically significant (Gautam et al., 2018a; Roy et al., 2018).

#### Immunoblotting

For protein sample preparations, 500 mg of mammary gland tissue was weighed and thoroughly homogenized in RIPA lysis buffer and phenylmethylsulfonyl fluoride (PMSF). The amount of protein in each sample was quantified using the Bradford assay. After quantification, proteins were separated on a 12.5% sodium dodecyl sulfate–polyacrylamide gel electrophoresis (SDS–PAGE) gel using the principle of Laemmle. In the subsequent step, proteins separated on the gel were transferred onto a polyvinylidene difluoride (PVDF) membrane (IPVH 00010, Millipore, Bedford, MA, United States). Proteins were transferred onto PVDF membranes with the Turbo Transfer assembly (Bio-Rad) operated at 25 V and 2A for 15 min at 16°C. The proteins transferred to the membrane were blocked for 3 h with a mixture of 5% BSA and 5% nonfat skimmed milk prepared in TBST, followed by incubation at 4°C with primary antibodies [SREBP-1c (SC-13551), HIF-1α (SC-13515), FASN (SC-55580), and (PHD-2 (SC-67030)] overnight. β-Actin was used as the standard reference. After overnight incubation with the primary antibody, the PVDF membrane was washed three times with TBST and then incubated with horseradish peroxidase (HRP)-conjugated secondary antibodies [anti-mouse (SC-31430, Pierce Thermo Scientific, USA), anti-rabbit (SC-2030), and anti-goat (SC-2020)] at room temperature for 3 h (Yadav et al., 2020). Finally, the membrane was washed once with TBST, and protein blots were developed and analyzed using ChemiDoc XRS+ (Bio-Rad).

## Statistical Analysis

The results were analyzed using the GraphPad Prism software (version 5.02). The values are presented as mean ± SD, and the statistical significance was calculated by one-way ANOVA followed by the Bonferroni test. Values of ^∗^*p* < 0.05, ^∗∗^*p* < 0.01, and ^∗∗∗^*p* < 0.001 were considered statistically significant.

## Results

### Effect of Drugs and Toxicants Upon Hemodynamic Profile

After establishing the cardiotoxic effect of doxorubicin in post-clinical studies, assessing the cardiotoxic effect of the drug has become important (Zhao and Zhang, 2017). Therefore, first of all, we recorded the ECG of experimental animals in order to evaluate the hemodynamic profile after treatment. Hemodynamic profiles were measured using ECG (Figure 1B) and HRV (Table 1). The results were analyzed online by MetaboAnalyst by selecting PCA from chemometric analyses. The 2D score plot of PCA of NC and treatment groups showed excellent separation (not shown in the diagram). The DMSO-treated group moved away from the NC (G1) group, indicating that DMSO administration somehow affected cardiac activity. DMBA administration displaced the G2 control group.

**TABLE 1:** Effect of VOA and VIN on HRV of experimental animals.

Groups	G1	G2	G3	G4	G5	G6	G7
**Time domain**							
Average RR	234.45 ± 22.98	162.35 ± 0.070	244.35 ± 0.07***	154.25 ± 5.30	186.4 ± 4.94**	147.75 ± 4.94	195.9***
Median RR	235 ± 22.62***	163 ± 0.00	246 ± 0.00***	154.5 ± 4.94	185 ± 1.41*	148 ± 1.41	194.5 ± 14.84***
SDRR	3.806 ± 1.66	234.45 ± 22.98***	6.465 ± 0.03	1.40 ± 0.50*	9.145 ± 7.29	1.9 ± 0.71	5.075 ± 0.40
CVRR	0.016 ± 0.00*	0.0465 ± 0.00	0.024 ± 0.002	0.0085 ± 0.00**	0.0475 ± 0.038	0.012 ± 0.004*	0.0255 ± 0.00
Average Rate	257.25 ± 25.24***	370.4 ± 0.14	245.7 ± 0.00***	388.3 ± 11.87	322.9 ± 7.35***	406.15 ± 3.747**	307.25 ± 21.99***
SD Rate	4.465 ± 2.77***	17.635 ± 1.43	3.158 ± 0.031***	3.525 ± 1.08***	15.65 ± 11.74	5.41 ± 2.149**	7.94 ± 0.48*
**Frequency domain**							
VLF Band	28.425 ± 8.56	30.11 ± 4.31	54.61 ± 2.85*	39.63 ± 17.77	70.43 ± 25.90***	30.67 ± 1.96	64.52 ± 5.71***
LF	10.27 ± 10.13	11.64 ± 2.22	2.64685 ± 1.23	7.045 ± 4.27	11.271 ± 9.20	5.511 ± 0.12	5.09 ± 2.51
HF	44.025 ± 7.67	43.24 ± 3.37	28.995 ± 1.025*	41.265 ± 6.17	15.9435 ± 14.51***	48.505 ± 1.80	23.845 ± 0.37***
LF/HF	0.2156 ± 0.19	0.2795 ± 0.07	0.089 ± 0.04	0.164 ± 0.07	0.75 ± 0.11***	0.111 ± 0.001	0.21 ± 0.09

All values represent Mean ± SD. Comparisons were made on the basis of one-way ANOVA followed by Bonferroni test. All groups were compared to the DMBA treated group. Values *p < 0.05, **p < 0.01, and ***p < 0.001 were considered significant. G1-Normal control (normal saline 3ml/kg,o.p.),G2-Toxic control (DMBA 8 mg/kg, i.v.), G3-VOA 1mg/kg, s.c.), G4-(VIN 1 mg/kg, i.v.), G5-(VOA 0.5 mg/kg, s.c.+ VIN 0.5 mg/kg, i.v.), G6-(VOA 1 mg/kg, s.c.+ VIN 1 mg/kg, i.v.) G7DMSO 3%, 2 ml/kg,s.c.

Treatment with monotherapy with VOA (G3) and VIN (G4) further displaced NC. However, treatment with combination therapy displaced G5 and G6 far from NC (G1), indicating the cardiac toxicity of the drug. To further confirm the cardiotoxicity of the drugs, data were again analyzed using a box-cum-whisker plot (Figure 1C). A significant difference in R and P amplitudes was observed in G2 compared to the NC and treatment groups. P amplitude increased in all the groups compared to the NC group, with a more pronounced effect in the VIN-treated group. The P-wave duration significantly increased with VOA (G3) but remained the same in all the other groups. Compared to that in the standard control, the PR interval increased with high-dose monotherapy of VOA, VIN, and high-dose combination therapy. The QT interval was observed to be decreased in DC, in combination with low and high doses in G2, whereas it was observed to increase with monotherapy (G3 and G4). A higher R amplitude was observed in G2 than in the other treatment groups. S amplitude was decreased in DC, G3, and G6, while it was increased in the VIN high-dose treatment group. The T amplitude decreased with DMSO treatment but increased with a high dose of monotherapy. A slight decrease in HR was observed in the DMSO-treated group, but a sharp decrease in HR was observed with DMBA administration in G2. HR was even more decreased in all treatment groups (G3, G4, G5, and G6) with the institution of therapy when compared to NC, DC, and G2. The JT interval increased with monotherapy, but the RR interval was observed in all treatment groups. The Q amplitude increased with the high VIN dose, but no change was observed with other treatments. The T peak was significantly increased with a high dose of combination therapy, but a slight increase in the T peak was also observed with monotherapy.

### Effect of Voacamine and Vincristine Upon Morphological Changes

Morphological examination of tissue through microscopic techniques like carmine staining and histopathology can provide valuable information about the drug’s effect on the target tissue. It can also help in deciding the stage and severity of the disease. Therefore, we performed the carmine staining and histopathology of mammary gland tissue and vital organs like the liver and kidney in our next experiment.

### Mammary Gland Whole-Mount Staining

**FIGURE 2:**
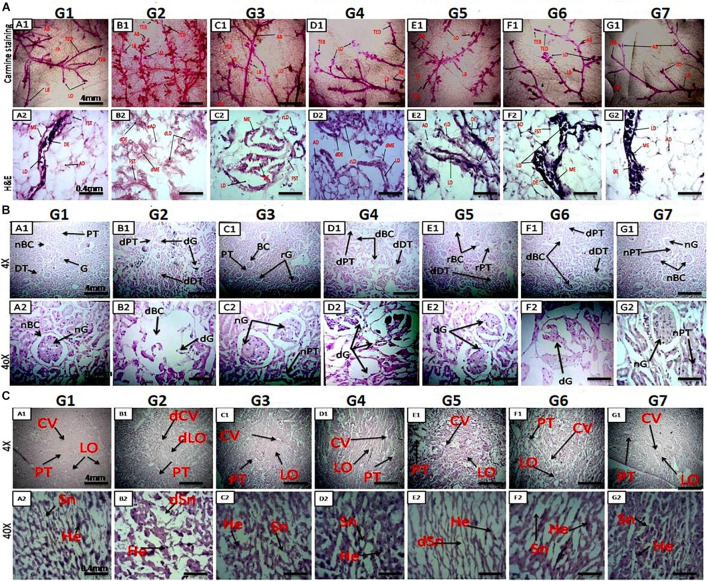
**(A)** Microscopic examination of rat mammary gland tissue through carmine staining (A1-NC) and H&E staining (A2–G2). A1 and NC show that carmine staining of the mammary gland tissue of NC- and DMSO-treated groups had fewer alveolar buds (ABs), lobules (LOs), lateral buds (LB), and terminal end ducts (TEDs). (B1) DMBA-treated G2 group showing extensive branching of TED, high numbers of AB and LB, and large numbers of LO. (C1–F1) Mammary gland of rats treated with monotherapy and combination therapy of VOA (1 mg/kg) and VIN (1 mg/kg), showing fewer AB counts, LB, TED, and LO when compared to G2. (A2,G2) H&E staining of the mammary gland tissue of the NC group showing typical architecture of the lactiferous duct (LD), adipose tissue (AT), myoepithelial tissue (ME), ductal epithelium (DE), and fibrous stromal tissue (FST). (B2) H&E staining of the mammary gland tissue of the DMBA-treated group showing distorted architecture of the lactiferous duct (dLD), damaged myoepithelium (DME), and damaged ductal epithelium (dDE). (C2–F2) Treatment with monotherapy and combination therapy of VOA (1 mg/kg) and VIN (1 mg/kg) significantly protected against further damage to the mammary tissue, as evidenced by the restoration of regular architecture of LD, AT, and DC ME. **(B)** Effect of VOA and VIN therapy on rat kidney. (A–G) Kidney sections of rats treated with VOA and VIN at × 4 and × 40 magnification. (A) Kidney of normal rats showing standard architecture of BC, G, PCT, and DCT. (B) G2 rat kidney showing DBC, dG, and distorted architecture of PCT and DCT. (C) VOA-treated rat kidney showing typical architecture of BC, G, PCT, and DCT. (D) VIN-treated rat kidney showing extensive damage to BC, G, PCT, and DCT. (E, F) Kidney of rats treated with combination therapy of VOA and VIN showing even more damage to BC, G, and microtubules (PCT and DCT). (G) Kidney of rats treated with 3% DMSO showing standard architecture of the kidney. dBC, damaged Bowman’s capsule; dG, damaged glomerulus; PCT, proximal convoluted tubule; DCT, distal convoluted tubule. **(C)** Effect of VOA and VIN therapy on rat liver. A1-NC and A2–G2: Histology of rats treated with VOA and VIN at × 4 and × 40. (A1,A2) Liver sections of NC rats showing typical architecture of liver lobules (LO), hepatocytes (He), sinusoids (Sn), and central vein (CV). (B1,B2) Liver section of G2 rats showing dilated central vein, damaged He, and distorted Sn and Lo. (C1,C2) Liver sections of VOA-treated rats showing slight dilation of CV, intact lobular structure, no distortion of Sn, and regular He. (D1,D2) Liver section of VIN-treated rats showing dilated CV, distended portal triads (PT), and enhanced sinusoidal space. (E1,E2,F1,F2) Liver sections of rats treated with low- and high-dose combination therapy of VOA and VIN showing extensive distortion of Sn, damage to He, enlargement of central and portal triads, and disruption of lobular structure. (NC-G2) Liver section of rats treated with 3% DMSO showing standard liver architecture similar to normal.

Carmine staining was used to analyze angiogenesis in malignant tumors. Single-dose administration of DMBA caused a significant increase in angiogenesis, marked by an increase in ABs and LO in G2 compared to the NC group (Figures 2A-A1–G1). Administration of monotherapy and combination therapy (low and high doses) of VOA and VIN inhibited angiogenesis in the experimental animals. Moreover, the results showed that VOA alone (G3) and combination therapy resulted in better angiogenesis inhibition than the VIN (G4) treatment, characterized by a lower number of AB counts and LO.

### H&E Staining of Sectioned Mammary Glands

H&E staining was performed to visualize the microscopic architecture of the mammary gland tissue of control and toxic-treated rats. Upon DMBA administration, significant damage to the lactiferous duct (LD), adipocytes (ADs), and distorted myoepithelium (ME) and ductal epithelium (DE) were observed in G2 when compared to the NC group (Figures 2A-A2–G2). Initiation of therapy with VOA and VIN (as monotherapy and combination therapy) provided significant protection to the mammary gland tissue, as evidenced by the regeneration of LD, fibrous stromal tissue (FST), and ADs. A better improvement than expected was observed with the combination therapy. Cellular damage was not observed in the DMSO (3%)-treated animals. This further suggests that DMSO at this concentration has no side effects if used as a solvent.

### H&E Staining of Liver and Kidney Tissue

H&E staining of rats treated with DMBA caused significant damage to Bowman’s capsule (BC) and the glomerulus (G) (Figure 2B). The space between BC and G was observed to be increased, and the structure of proximal convoluted tubules (PCTs) and distal convoluted tubules (DCTs) was observed to be primarily distorted in G2 compared to those in the NC (Figures 2B-B1,B2). The typical architecture of the renal tissue was observed in the VOA-treated group. However, treatment with monotherapy with VIN caused significant damage to BC and G and the destruction of microtubules (PCT and DCT) (Figures 2B-D1,D2). Extensive damage of BC, G, PCT, and DCT was observed in groups treated with VOA and VIN combination therapy (Figures 2B-E1,E2,F1,F2). The standard architecture of renal tissue was observed in the DMSO-treated group.

Liver histology of DMBA-treated G2 showed damage to liver sinusoids, dilation of the central vein, and resultant disruption of the lobular architecture compared with the normal (Figures 2C-B1,B2). Relatively minor destruction of the liver architecture was observed with monotherapy of VOA and VIN (Figures 2C-C1,C2,D1,D2). However, extensive damage to the lobular structure was observed with both low- and high-dose combination therapy, as evidenced by considerable damage to hepatocytes, sinusoids, and dilatation of the central vein (Figures 2C-E1,E2,F1,F2). No damage to the liver structure was observed after DMSO administration.

### Effect of Antioxidant Markers Upon Mammary Gland Carcinoma

Single-dose administration of DMBA significantly increased the level of TBARS in G2 (0.65 ± 0.184 and 119.46 ± 6.58) when compared to the NC (0.13 ± 0.032 and 72.007^∗∗∗^ ± 6.64) groups (Table 2). Treatment with monotherapy of VOA and VIN worked well to keep the level of TBARS and PC to the minimum in treatment groups (G3: 0.27 ± 0.20^∗∗∗^, G4: 0.32 ± 0.118^∗∗∗^, G5: 0.138 ± 0.046^∗∗∗^, G6: 0.17 ± 0.017^∗∗∗^, and G3: 67.87 ± 4.76^∗∗∗^, G4: 41.29 ± 10.66^∗∗∗^, G5: 67.38 ± 10.93^∗∗∗^, and G6: 44.92 ± 12.40^∗∗∗^). Interestingly, treatment with a combination of low-dose therapy provided a better improvement in TBARS, whereas, in the case of PC, better restoration of activity was observed with VOA monotherapy and low combination dose. GSH, SOD, and catalase are enzymes that work together to neutralize the harmful reactive oxygen species (ROS) generated in the body. The levels of SOD, catalase, and GSH were significantly decreased after DMBA administration in G2 (GSH: 0.12 ± 0.005, SOD: 0.014 ± 0.002, and catalase: 0.29 ± 0.064) when compared to the NC (GSH: 0.36 ± 0.002, SOD: 0.058 ± 0.006, and catalase: 0.72 ± 0.004) groups. As shown in Table 1, the levels of all three enzymes significantly increased after treatment with monotherapy and combination therapy with VOA and VIN. Combination therapy also resulted in improved antioxidant activity.

**TABLE 2:** Effect of VOA/VIN on oxidative stress markers.

Groups	TBARS (nM of MDA/μg of protein)	GSH (mg %)	SOD (Units of SOD/ /mg of protein)	Catalase (nM of H2O2/ /min/mg of protein)	Protein carbonyl (nM/ml unit)
G1	0.13 ± 0.032	0.36 ± 0.002	0.058 ± 0.006	0.72 ± 0.004	72.007*** ± 6.64
G2	0.65 ± 0.184	0.12 ± 0.005	0.014 ± 0.002	0.29 ± 0.064	119.46 ± 6.58
G3	0.27 ± 0.20***	0.25 ± 0.030	0.034*** ± 0.002	0.51 ± 0.123***	67.87 ± 4.76***
G4	0.32 ± 0.118***	0.21 ± 0.002***	0.041 ± 0.009***	0.45 ± 0.08***	41.29 ± 10.66***
G5	0.138 ± 0.046***	0.27 ± 0.001***	0.039 ± 0.002***	0.53 ± 0.084***	67.38 ± 10.93***
G6	0.17 ± 0.017***	0.29 ± 0.008***	0.048 ± 0.001***	0.57 ± 018***	44.92 ± 12.40***
G7	0.13 ± 0.046***	0.39 ± 0.007***	0.059 ± 0.001***	0.77 ± 0.004***	75.25 ± 3.11***

All values represent mean ± SD. Comparisons were made on the basis of one-way ANOVA followed by Bonferroni test. All groups were compared to the DMBA treated group. Values ***p < 0.001 were considered significant. NC-Normal control (normal saline 3 ml/kg,o.p.), TC-Toxic control (DMBA 8 mg/kg, i.v.), T1-Treatment 1 (VOA 1 mg/kg, s.c.), T2-Treatment 2 (VIN 1 mg/kg, i.v.), T3-Treatment 3 (VOA 0.5 mg/kg, s.c.+ VIN 0.5 mg/kg, i.v.), T4-Treatment 4 (VOA 1 mg/kg, s.c.+ VIN 1 mg/kg, i.v.), DC-Dummy control (DMSO 3%).

### ^1^H-NMR Method for Serum Metabolite Profiling

**FIGURE 3:**
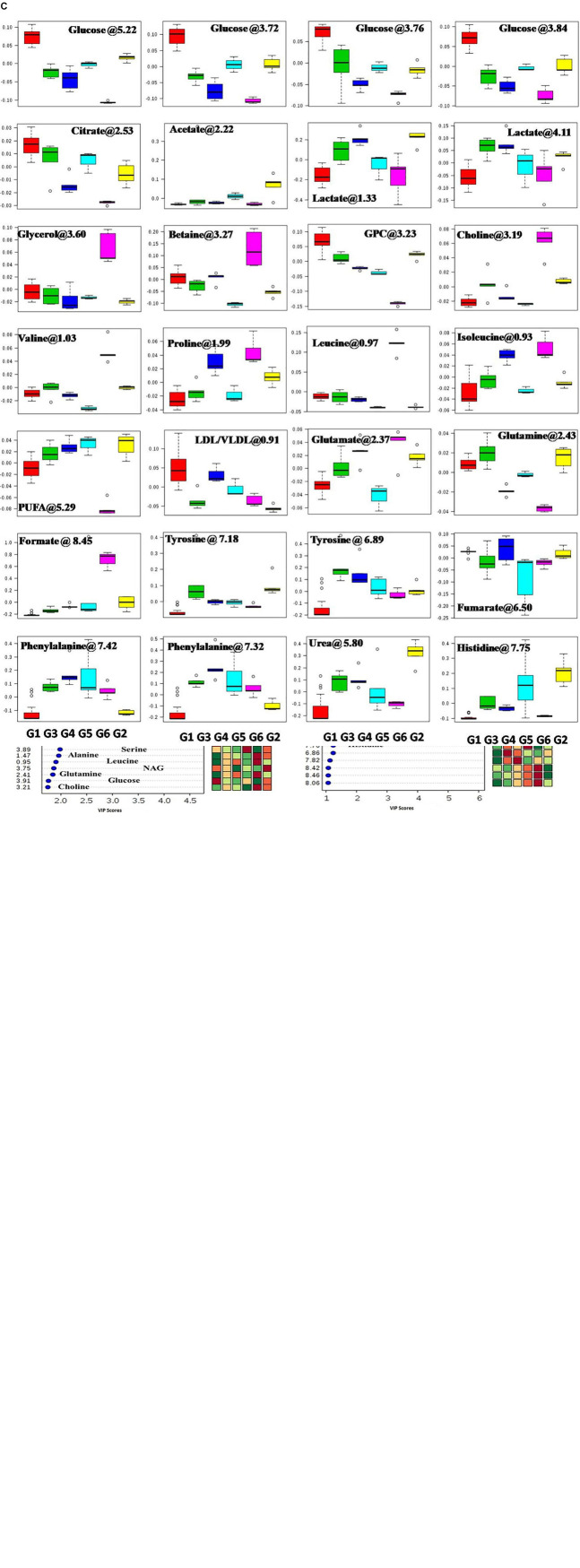
**(A)** Stock plot representation of 1D-H-NMR of serum metabolites of experimental rats. NMR spectra of serum samples are represented in distinguishing color formats (A– F shown in the above figure). The peaks annotated in the figure below show the assignment of serum metabolites. The abbreviations used are as follows: LDL/VLDL, low/very-low-density lipoproteins; PUFA, polyunsaturated fatty acids; BCAA, branch chain amino acids; Leu, leucine; NAG, *N*-acetyl glycoproteins; Arg, arginine; Lys, lysine; m-I, myo-inositol; GPC, glycerol phosphocholine; and glucose resonances are indicated using an asterisk (^∗^). **(B)** 2D-PLS-DA score plot of rat serum metabolites. The combined 2D-PLS-DA score plots between the groups clearly indicated that the DMBA-induced metabolic changes were essentially reset to their average levels after voacamine and vincristine treatment in monotherapy and with combined therapy of both drugs, as inferred by the decreased separation between the treated and regular control groups. (A) The complete CPMG data matrix was used for PLS-DA modeling; (B) the upfield spectral region (from 5.4 to 8.6 ppm, aromatic region) was excluded from the data matrix to evaluate the discriminatory significance of aromatic residues. The 10-fold validation parameters (R2, Q2, and accuracy) for the resulting PLS-DA model are shown in the inset. **(C)** Box-cum-whisker plot of rat serum metabolites. Representative box-cum-whisker plots showing quantitative variation in relative signal integrals for serum metabolites. For the presented metabolite entities, the VIP score is > 1, and statistical significance is at the level of *p* ≤ 0.05. In the box plots, the boxes denote the interquartile ranges, the horizontal lines inside the boxes denote the median, and the bottom and top boundaries of the boxes are the 25th and 75th percentiles, respectively. The lower and upper whiskers are the 5th and 95th percentiles, respectively.

**FIGURE 4:**
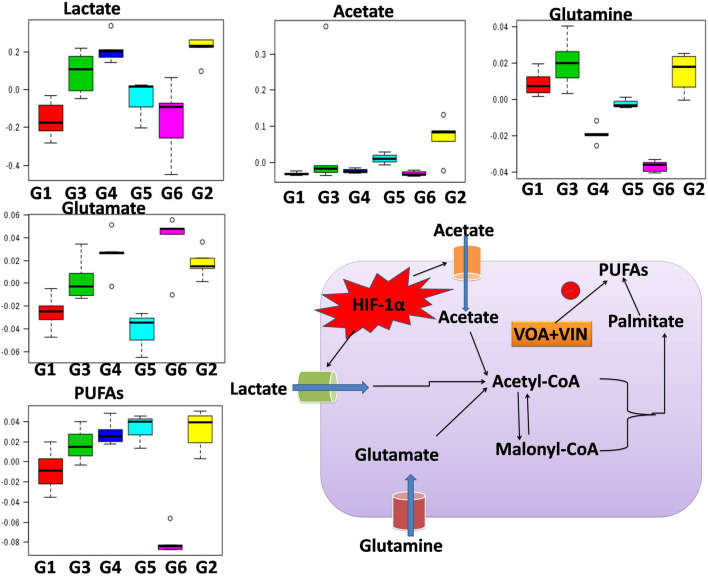
Hypoxia alters metabolic reprogramming in cancer cells to enhance fatty acid synthesis. HIF-1α shifts the metabolism of glucose through glycolysis, which fuels the fatty acid pathway. Also, glucose-derived citrate is insufficient to fulfill the heavy acid demand. Therefore, under hypoxia, cancer cells utilize abnormal metabolites like acetate, lactate, and glutamate to fuel the fatty acid pathway.

Since disease-affected organ tissues or cells produce abnormal metabolites which are washed away by the interstitial fluid and accumulate in the blood plasma/serum, serum metabolic profiles can give vital information about the diagnosis of a disease like cancer. Therefore, serum samples from all the grouped animals were collected at the end of the study. The collected serum samples were analyzed by ^1^H-NMR to predict changes in serum metabolomics, and the results are presented in Figures 3A–C, 4. A total of 27 markers, namely Isoleucine, Leucine, Valine, Lactate, Alanine, Acetate, NAG, Glutamate, Glutamine, Citrate, Proline, Choline, GPC, Glycine, Glycerol, Betaine, Glucose, a-Glucose, b-Glucose, Serine, PUFA, Urea, Tyrosine, Phenylalanine, Histidine, Formate, and LDL/VLDL (Table 3). Significantly higher levels of glucose, lactate, acetate, glutamate, fumarate, and PUFAs were significantly higher in G2 than in the normal group. A significant reduction in aforesaid metabolites was observed in the treatment groups with the institution of therapy. Acetate levels reverted to normal in all the treated groups, while lactate levels were significantly reduced by the combination therapy (favorably with higher dose combination). Monotherapy also worked well in lactate reduction, but no significant change in lactate levels was observed. The level of glutamate was reduced in G3, G4, and G5 but increased in G6. Glutamine remained unchanged in G3 but reduced in G4, G5, and, and G6. With regard to PUFAs, a linear reduction pattern was observed with monotherapy and combination therapy.

**TABLE 3:** List of cross analyzed metabolites.

S.No	Metabolites	ppm Values	G1 vs G2	G1 vs G3	G1 vs G4	G1vs G5	G1vs G6
1	Isoleucine	0.93 (t), 1.01 (d)	**↑**	**↑**	**↓**	**↓**	**↑**
2	Leucine	0.95 (d), 0.96 (d)	**↓**	**↑**	**↓**	**↓**	**↑**
3	Valine	0.98 (d), 1.04 (d)	**↑**	**↑**	**↓**	**↓**	**↑**
4	Lactate	1.33 (d), 4.12 (q)	**↑**	**↑**	**↑**	**↑**	
5	Alanine	1.46(d)	**↑**	**↑**	**↑**	**↑**	**↑**
6	Acetate	1.91 (s)	**↑**	**↑**	**↑**	**↑**	**↓**
7	NAG	2.04 (m)	**↑**	**↓**	**↓**	**↓**	**↓**
8	Glutamate	2.07(m), 2.34(m)	**↑**	**↑**	**↑**	**↓**	**↓**
9	Glutamine	2.11(m), 2.43(m)	**↑**	**↑**	**↓**	**↓**	**↑**
10	Citrate	2.53 (d), 2.69 (d)	**↓**	**↓**	**↓**	**↓**	**↓**
11	Proline	2.01(m), 1.99(m)	**↑**	**↑**	**↑**	**↑**	**↑**
12	Choline	3.20 (s), 4.02(m)	**↑**	**↑**	**↑**	**↓**	**↑**
13	GPC	3.228(s), 4.34(m)	**↓**	**↓**	**↓**	**↓**	**↓**
14	Glycine	3.55 (s)	**↓**	**↓**	**↓**	**↓**	**↓**
15	Glycerol	3.56, 3.65 (d)	**↓**	**↓**	**↓**	**↓**	**↑**
16	Betaine	3.268	**↓**	**↓**	**↓**	**↓**	**↑**
17	Glucose	3.24(t), 3.53(q) 3.49(t), 3.71(t) 3.40(t), 3.41(t) 3.46(m), 3.83(m) 3.72(q), 3.76(q), 3.84(q), 3.90(q)	**↓**	**↓**	**↓**	**↓**	**↓**
18	α-Glucose	4.65(d)	**↓**	**↓**	**↓**	**↓**	**↓**
19	β-Glucose	5.23(d)	**↓**	**↓**	**↓**	**↓**	**↓**
20	Serine	3.94, 3.98 (tt)	**↑**	**↓**	**↓**	**↑**	**↓**
21	PUFA	5.29	**↑**	**↑**	**↑**	**↑**	**↓**
22	Urea	5.80	**↑**	**↑**	**↑**	**↑**	**↑**
23	Tyrosine	6.89 (d), 7.18 (d)	**↑**	**↑**	**↑**	**↑**	**↑**
24	Phenylalanine	7.32(d), 7.35(t) 7.42 (d)	**↑**	**↑**	**↑**	**↑**	**↑**
25	Histidine	7.05 (s), 7.76 (s)	**↑**	**↑**	**↑**	**↑**	**NA**
26	Formate	8.45 (s)	**↑**	**↑**	**↑**	**↑**	**↓**
27	LDL/VLDL	0.88, 0.91,	**↓**	**↓**	**↓**	**↓**	**↓**

*The list of metabolites responsible for variation and class separation between normal control (G1) vs toxic control (G2) and Normal control vs treatment groups (G3, G4, G5 and G6). The metabolic biomarkers were screened based upon the VIP score values > 1.0 (derived from PLS-DA modeling, showing discrimination significance) and then tested (using univariate and student t-test) for statistical significance based on p-value < 0.05. The up (**↑**) and down (**↓**) arrows represent, respectively, increased and decreased metabolite levels within the groups compared to controls.*

### Effect of Voacamine and Vincristine Upon Fatty Acid Methyl Ester Analysis

**FIGURE 5:**
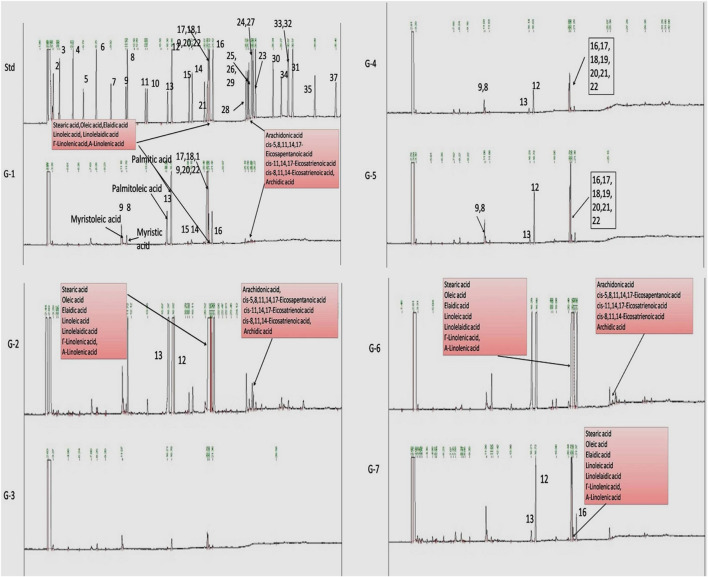
Effect of VOA and VIN on fatty acid composition of mammary gland tissue. Std, standard (Supelco FAME 37 component, Sigma-Aldrich); NC, normal control (saline 2 ml/kg); G2, toxic control (DMBA 8 mg/kg, i.v.); G3, voacamine (VOA) (1 mg/kg, s.c.); G4, vincristine (VIN) (1 mg/kg, i.v.); G5, VOA (0.5 mg/kg, s.c.) + VIN (0.5 mg/kg, i.v.); G6, VOA (1 mg/kg, s.c.) + VIN (1 mg/kg, i.v.); and G7, DC-DMSO 3% (2 ml/kg).

Gas chromatography is a versatile technique to study the fatty acid profile of a target tissue or sample. Also, tissues or cells produce different types of monounsaturated fatty acids (MUFAs) and PUFAs in normal and diseased conditions. Therefore, next, we chose to perform GC-ID analysis of mammary gland tissue of experimental animals. FAME analysis of mammary gland tissue of control and treatment groups was analyzed by GC-FID as mentioned in the Materials and Methods section. A significant change in the fatty acid composition of G2 animals was observed compared to that of NC. Higher levels of PUFAs were observed in G2 after DMBA administration. Upon treatment with VOA and VIN monotherapy, as well as with combination therapy, a significant reduction in the synthesis of PUFAs was noted (Figure 5).

### Immunoblotting Analysis

**FIGURE 6:**
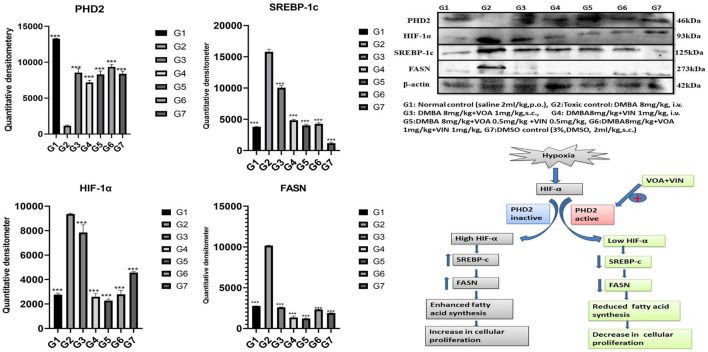
Effect of VOA and VIN on hypoxic markers and fatty acid synthesis markers. Immunoblotting of the respective group [NC, normal control (saline 2 ml/kg); G2, toxic control (DMBA 8 mg/kg, i.v.); G3, voacamine (VOA) (1 mg/kg, s.c.); G4, vincristine (VIN) (1 mg/kg, i.v.); G5, VOA (0.5 mg/kg, s.c.) + VIN (0.5 mg/kg, i.v.); G6, VOA (1 mg/kg, s.c.) + VIN (1 mg/kg, i.v.); and DC-DMSO 3% (2 ml/kg)] for HIF-1α, PHD-2, SREBP-1c, and FASN concluded the hypoxic microenvironment after DMBA administration. Values are presented as the mean ± standard deviation. Comparisons were made using one-way ANOVA followed by Bonferroni multiple test. All groups were compared to the DMBA-treated group (***p < 0.001). Please note that the blots were cropped horizontally from the same gel at different time intervals.

**FIGURE 7:**
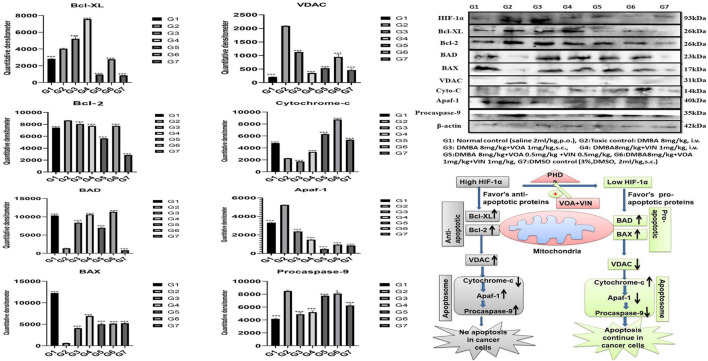
Effect of VOA and VIN on mitochondrial apoptotic markers. Immunoblotting of the respective group [NC, normal control (saline 2 ml/kg); G2, toxic control (DMBA 8 mg/kg, i.v.), G3, voacamine (VOA) (1 mg/kg, s.c.); G4, vincristine (VIN) (1 mg/kg, i.v.); G5, VOA (0.5 mg/kg, s.c.) + VIN (0.5 mg/kg, i.v.); G6, VOA (1 mg/kg, s.c.) + VIN (1 mg/kg, i.v.); and DC-DMSO 3% (2 ml/kg)] for Bcl-XL, BAD, BAX, cytochrome c, caspase 9, Apaf-1, and VDAC concluded the mitochondrial-mediated death apoptosis after DMBA administration. Values are presented as the mean ± standard deviation. Comparisons were made using one-way ANOVA followed by Bonferroni multiple test. All groups were compared to the DMBA-treated group (*p < 0.05, ***p < 0.001). Please note that the blots were cropped horizontally from the same gel at different time intervals.

Western blotting is one of the most reliable molecular biology techniques to study the effect of drugs and toxicants on the expression of a protein molecule. It is also evident that the expression of various proteins is impaired in various disease conditions like cancer. Therefore, mammary gland tissue was collected, and the protein of hypoxic pathways like HIF-1, SREBP-1c, FASN, and PHD was quantified and analyzed. Both combination and monotherapy with VOA and VIN suppressed the expression of HIF-1α, SREBP-1c, and FASN. Monotherapy with VOA (G3) 1 mg/kg caused a significant reduction in HIF-1α, FASN, and SREBP-1c levels compared to those in the toxic group, while at the same time, PHD-2 levels were increased. Similarly, VIN 1 mg (G4) also provided better inhibition of HIF-1α, FASN, and SREBP-1c compared to VOA 1 mg (Figure 6). Combination therapy of VOA and VIN, both low (G5) and high doses (G6), significantly reduced the expression of HIF-1α, SREBP-1c, and FASN compared to those in the toxic group. Interestingly, both doses of the combination therapy significantly enhanced PHD-2 expression. These results are inconsistent with those of *in silico* docking studies. In addition, better activation of PHD-2 was observed with the high-dose (G6) combination therapy. After DMBA administration, a significant change in the expression of anti-apoptotic (Bcl-XL) and pro-apoptotic (BAD and BAX) proteins was noted in toxic control animals. In addition, increased expression of VDAC, Apaf-1, and caspase 9 was observed in the toxic control group. Nevertheless, VOA and VIN therapy imparted significant protection and worked well to restore the changes (Figure 7).

## Discussion

PUFAs are crucial for plasma membrane synthesis in rapidly dividing cancer cells of mammary gland tissue, and dietary sources alone are insufficient to accomplish this (Yadav et al., 2018). Therefore, cancer cells must take over an alternative fatty acid synthesis pathway, and HIF-1α helps them to adapt to this (Bensaad et al., 2014). Previous studies have reported that HIF-1α acts in a thoughtful manner and modifies the tumor microenvironment in such a way that indirectly enhances the fatty acid synthesis required to synthesize the plasma membrane and furnish other purposes in cancer cells (Furuta et al., 2008). Considering the role of HIF-1α and FASN, the present study was undertaken to downregulate HIF-1α by activating PHD-2 with natural drugs VOA alone and in combination with VIN, and the findings of the study have been discussed in the preceding section.

Cardiac toxicity associated with anticancer drugs is pervasive. Therefore, for the analysis of cardiac toxicity, the hemodynamic profile of the animals was determined (Figure 1B and Table 1). Normal ECG and HRV were recorded in the NC group when the vehicle was administered for 3 months. DMSO is a universal solvent for hydrophobic drugs, and its cardiotoxic effects are well established (Kramer et al., 1995). DMSO administration decreased the HR and ST and T intervals and increased the RR and P amplitudes in the DMSO control group. A decrease in HRV after VIN treatment has already been reported in previous studies (Ilgin et al., 2018), and the same was also reflected in the present study. A significant increase in R amplitude was observed in the toxic control group after DMBA administration. It is reported that the R wave reflects the ventricular function, and the increase in R amplitude reflects severe dysfunction and severe coronary narrowing (Baron et al., 1980). This shows that DMBA has adverse effects on the heart, and the same is also reflected in the current study. The prolonged RR interval represents delayed conduction of the sinoatrial or SA nodal impulses to the ventricles and represents the first-degree AV block (Cooper and Tariq, 2017). The increase in RR interval groups was observed in all the groups that clearly marked the AV blockage action of therapy. While a monotherapy documented less AV blockage, both by VOA and VIN, their combination therapy caused even more increase in RR interval (G5 and G6), which indicates that combination therapy has a potentially toxic effect on the AV conduction mechanism and warns against their use in cardiac patients. Here, the author would like to the point that monotherapy with VOA (1 mg/kg, s.c., G3) caused minimal AV blockage, which points to its safety in cardiac patients. It is previously reported that prolonged T wave and interval in the resting ECG indicate that the patient is at increased risk of sudden cardiac death (Panikkath et al., 2011; Azarov et al., 2018). In the current study, a significant increase in T wave was also observed with a higher dose of combination therapy (G6), again evidenced by the cardiotoxic effect of combination therapy. Higher-dose combination therapy should be avoided as it can predispose patients to sudden cardiac death and myocardial infarction. The above findings are further supported by a significant drop in the HR of G5 and G6 animals. Previous studies have reported that a significant decrease in HR indicates coronary artery disease, heart attack, and infections such as endocarditis. Overall, it can be said that combination therapy of VOA and VIN should be avoided in patients with previous cardiac problems (Ho et al., 2011). Also, monotherapy of VOA imparts minimum cardiac toxicity. Here, the concomitant administration of VOA with VIN/DMSO further exacerbated the cardiotoxic effect (Figure 1C).

After assessing the cardiac toxicity of the given therapy, we next performed the microscopical examination of mammary gland tissue by carmine staining and histopathology. Carmine staining is a vital staining technique to evaluate the anti-angiogenetic effect, while histopathological examination can help in the detection of cellular damage. Studies have reported that hypoxia in solid tumors plays an essential role in angiogenesis by increasing the levels of oxygen and other nutrients. Various studies have reported that increased levels of HIF-1α stimulate angiogenesis in tumor cells (Sonveaux et al., 2007, 2012). In the present study, results of the carmine staining showed an increase in the AB count and LOs in the toxic control group, which depicts the formation of neovascularization (Figure 2A-A1-NC) after DMBA administration. However, treatment with monotherapy and combination therapy of VOA and VIN reduced the AB count and LOs in the experimental animals, indicating suppression of angiogenesis after initiation of therapy. Therapy with VOA and VIN might regulate the HIF-1α pathway to abolish angiogenesis.

Histopathology of the mammary gland tissue of carcinogen-treated rats has shown extensive damage to the LD, AD, DE, and ME, as reported in previous studies (Hoshino et al., 2007). Extensive damage to the microarchitecture of mammary tissue, such as LD, AD, DE, and ME, was observed after DMBA administration in G2 control (Figures 2A-A2–G2). In addition, the opposite effect upon the LD, AD, DE, and ME was observed with VOA and VIN treatments, which evidenced the protective effect of the therapy. This indicates that VOA and VIN work at the cellular level to stop tumor progression. Histopathological examination of the mammary gland tissue further demonstrated the protective action of the therapy.

After performing the microscopy of mammary gland tissue, we carried out histopathology of the liver and kidney tissue in order to assess the liver and kidney toxicity. The kidney is a vital and sensitive organ in the body and is responsible for the filtration and excretion of metabolized drugs. Continuous filtration and excretion of harmful drug molecules, especially those belonging to the anticancer class, can cause injury to the nephrons of the kidney and ultimately cause nephrotoxicity if the dose is not monitored in a timely manner. Most anticancer drugs are reported to have varying degrees of nephrotoxicity in patients taking them (Małyszko et al., 2017). Nephrotoxicity due to chemotherapeutics is marked by necrosis of the epithelial lining of PCT and DC and injury to the BC (Perazella, 2012). Histopathological examination of the mammary glands of treated rats revealed the same type of damage to the microarchitecture of the kidney after DMBA administration (Figure 2B). Subsequent treatment with VIN monotherapy further exacerbated renal toxicity, as evidenced by the more significant damage to the PCT, DCT, and BC, as its nephrotoxicity has already been reported (Aitchison et al., 1990). It is worth mentioning that no remarkable change in the microarchitecture of BC, glomerulus, PCT, and DCT was noted; BC was noted in rats treated with VOA monotherapy, which confirms its safety in renal failure. Even more damage to the kidney was exhibited by both low and high doses of combination therapy marked by renal tubular epithelial cell necrosis, a loop of Henle, and glomerulus attributed to the high drug accumulation, in either of the two. Since VOA are known to have a Pgp (efflux pump) inhibitor (Condello et al., 2014), these might have helped intracellular pooling of VIN in the nephrons of experimental animals and could be a possible cause of nephrotoxicity (Figure 2B).

The liver is the site where most drugs undergo first-pass metabolism (except for those administered through the parenteral route; Maor and Malnick, 2013). Normal liver function is affected by continuous exposure to the high concentration of cytotoxic substances, which then leads to liver failure in some patients. Liver toxicity is marked by dilation of the central vein, damage to hepatocytes (He), distorted sinusoids (Sn), and LO, which are very evident in DMBA-treated animals (Figure 2C). Monotherapy with VOA and VIN worked well to prevent further damage to the liver of experimental animals, which documents the liver safety of both drugs at a given dose. However, the histology of combination therapy-treated rats showed considerable damage to the liver tissue, proving the VIN/VOA accumulation, or either of the two into the hepatocytes, consequently resulting in hepatotoxicity.

As microscopical examination evidenced the excessive damage to the mammary gland tissue, we further performed a biochemical analysis of mammary gland tissue in order to explore the role of free radicals in cellular destruction. Free radicals have been reported to play an essential role in the pathogenesis of cancer as well by causing genetic instability. In fact, most of the carcinogens exert their carcinogenic effect by generating free radicals like DMBA. These free radicals have the potential to introduce damages in the cellular DNA and consequently origin of cancer (Pourahmad et al., 2016). Biochemicals like TBARS, PC, and GSH are sensitive markers to evaluate antioxidant mechanisms active in the cells. Previous studies have reported the dysregulation of the antioxidant mechanism in mammary gland carcinoma cells (Ríos-Arrabal et al., 2013). Therefore, the evaluation of antioxidant machinery in the mammary gland tissue of experimental animals is critical. Various studies have reported the role of ROS in cancer, manifested by an increase in TBARS and PC and a reduction in the activity of SOD, GSH, and catalase (Tiwari et al., 2016; Al-Saeedan et al., 2018). The same type of antioxidant marker was also observed in the DMBA-treated rats. Interestingly, both monotherapy and combination therapy effectively restored TBARS, SOD, and other associated antioxidant markers. From this, we can expect that restoration of antioxidant activity could be one possible mechanism behind the anticancer potential of the drug (Table 2).

Numerous studies have reported that numerous changes occur in a biological system under disease conditions, which can be detected in biological fluids such as blood serum. To this end, a metabolic study was carried out to extract biomarkers and to understand the interplay between molecular and cellular components (Figures 3A–C; Shang and Wang, 2013; Hu and Sun, 2018). Previous studies have demonstrated that hypoxic tumor cells utilize an increasing amount of glucose to meet their energy requirements for biomass accumulation (Netea-Maier et al., 2018; Sormendi and Wielockx, 2018). Several studies have reported that tumor cells produce a high amount of lactate and glutamate that impart benefits to the tumor cells in various ways, such as fatty acid biosynthesis, immune protection, angiogenesis, and invasiveness (Palazon et al., 2017). The metabolic profile of DMBA-treated rats showed a high level of lactate, acetate, and glutamate and that of PUFAs, which is in accordance with previous studies; that is, excess lactate/glutamate is converted into fatty acids. The decreased glucose levels further confirmed these findings.

Interestingly, the reverse chronological order of the above metabolites was observed with VOA and VIN (monotherapy and combination therapy); that is, decreased levels of acetate, lactate, glutamate, glutamine, and PUFAs were noted in all the treatment groups. It is worth mentioning that monotherapy with VOA and high-dose combination therapy of VOA (1 mg/kg) and VIN (1 mg/kg) provided much better fatty acid synthesis inhibitory action than did VIN monotherapy and the low combination dose. Since cancer cells remain in high demand for fatty acids, glycolysis alone is insufficient to fulfill this requirement. Therefore, under hypoxia, cancer cells use abnormal metabolites such as lactate, acetate, and glutamate to feed the fatty acid synthesis pathway in cancer cells (Metallo et al., 2012; Gao et al., 2016; Lund et al., 2018). Serum metabolomics analysis of the present study established a relationship between glycolysis, acetate, lactate, and glutamate in fatty acid synthesis production, which is in line with previous findings (Figure 4).

As serum metabolomics analysis established the direct relationship between lactate and PUFAs, we further performed the FAME analysis of mammary gland tissue homogenate by GC. Interestingly, as expected, higher levels of PUFAs like arachidonic acid, *cis*-5,8,11,14,17-eicosapentaenoic acid, *cis*-11,14,17-eicosatrienoic acid, *cis*-8,11,14-eicosatrienoic acid, arachidonic acid, and linoleic acids were observed in toxic control (G2) animals when compared with the standard control (G1) animals (Figure 5). Also, reduced levels of PUFA were observed in treatment groups, which indicated that VOA and VIN therapy worked well to check PUFA synthesis in cancer cells. The groups having higher lactate levels also depicted higher levels of fatty acids. Previous studies have reported that hypoxic cancer cells remain in high demand of fatty acids required to make their plasma membrane along with the membrane of other cell organelles. *De novo* fatty acid synthesis alone cannot accomplish this requirement; therefore, cancer cells adopt some alternative mechanism and metabolites to meet their fatty acid demand (Corbet et al., 2016). Our finding with this study is consistent with those of previous studies and clearly evidenced that cancer can utilize lactate for fatty acid synthesis, and the same was inhibited by VOA and VIN combination and monotherapy.

Various studies have reported that HIF-1α works at the molecular level to reprogram fatty acid synthesis (Furuta et al., 2008; Munir et al., 2019). Therefore, we next performed western blotting of proteins involved in the hypoxia pathway like PHD-2, HIF-1α, SREBP-1c, and FASN. Several studies have confirmed that the expression of HIF-1α increases oxygen scarcity, which enhances the expression of other genes that indirectly benefit tumor cells in various ways (Maxwell et al., 1997; Palazon et al., 2017). A study conducted by Maxwell et al. on wild-type (wt) Hepa-1 cells and derivatives c4, c31, and Rc4 proved that HIF-1α plays a crucial role in GLUTs and VEGF (Maxwell et al., 1997). Another study conducted by Sun et al. on HeLa, HCG316, and cultured human primary epithelial cells showed that HIF-1α increased the levels of SREBP-1c and FASN (Sun et al., 2017). Intriguingly, a similar trend was also observed in the present study; that is, the levels of HIF-1α, SREBP, and FASN expression were upregulated while that of PHD-2 expression was downregulated after DMBA treatment (Figure 6). The opposite effect was observed after VOA/VIN therapy initiation. It has been reported that PHD-2 is a negative regulator of HIF-1α and that activation of PHD-2 alone can downregulate all its downstream effects (Chen et al., 2016). This affirms that VOA and VIN might have activated PHD-2, and subsequently, the level of HIF-1α along with FASN proteins would have dwindled as hypothesized.

Furthermore, to affirm whether hypoxia has any adverse effect on the proteins of the apoptotic pathway, proteins of the apoptotic pathway were also analyzed by western blotting. Several studies have reported that failure of apoptosis in normal cells is an indication of cancer transformation (Nagaraj et al., 2007; Elizabeth Besra et al., 2013; Sendoel and Hengartner, 2014). A decrease in anti-apoptotic proteins (Bcl-XL and Bcl-2) and an increase in pro-apoptotic proteins (BAD and BAX) indicate normal functioning of the mitochondrial apoptotic pathway (Ge et al., 2018). Immunoblotting showed an increase in the expression of Bcl-XL and a decrease in BAX and BAD proteins in the toxic control after DMBA administration. Furthermore, VDAC, Apaf-1, and caspase 9 were also found to be elevated, proving the failure of the mitochondrial apoptotic pathway. Treatment with monotherapy and combination therapy of VOA and VIN restored the apoptotic pathway in the treatment groups, as evidenced by the increase in the level of cytochrome c (Figure 7).

Overall, if the results of metabolomics, GC, and western blotting are taken together, it appears that DMBA induced mammary gland carcinoma cells, increasing acidity in the tumor microenvironment to enhance fatty acid synthesis. Under hypoxia, glycolysis mainly occurs in tumor cells, and excess lactate is pumped out in the tumor microenvironment, which creates a proton gradient across the membrane of cancer cells. Because of the proton gradient, protons are taken up by nearby cancer cells, which further decreases the intracellular pH in these cells (Corbet et al., 2016; Ward et al., 2020). Increased acidity activates the endoplasmic reticulum protein SREBP-1c, which translocates to the nucleus and enhances the expression of genes and enzymes involved in the fatty acid pathway. VOA and VIN therapy activate PHD-2, which leads to hydroxylation and proteolytic degradation of HIF-1α, thus reducing lactate-induced fatty acid synthesis.

## Conclusion

The authors would like to conclude that monotherapy with VOA (G3) and combination therapy with VIN can regulate HIF-1α-guided fatty acid synthesis in cancer cells by activating PHD-2 *in vivo*. The combination therapy of VIN and VOA can significantly reduce the progression of mammary gland carcinoma by inhibiting fatty acid synthesis. However, the proposed combination therapy offers a significant disadvantage of being hepatotoxic and nephrotoxic. As far as monotherapy of VOA (G3) is concerned, negligible cardiotoxicity, hepatotoxicity, and nephrotoxicity were observed along with significant inhibition of fatty acid synthesis. Further work should be carried out to delineate its role as a potent anticancer drug.

## Data Availability Statement

The original contributions presented in the study are included in the article/Supplementary Material, further inquiries can be directed to the corresponding author/s.

## Ethics Statement

The animal study was reviewed and approved by UIP/IAEC/May-2016/06.

## Author Contributions

LS performed the benchwork. SRo performed the carmine staining, histopathology, and western blotting. AK and SRa performed the biochemical estimation. DK supervised the NMR studies. MS performed the statistical studies, *in silico*, and compiled the data. GK perceived the idea, designed and supervised the whole study, and prepared and proofread the final manuscript. All authors contributed to the article and approved the submitted version.

## Conflict of Interest

The authors declare that the research was conducted in the absence of any commercial or financial relationships that could be construed as a potential conflict of interest.

## Publisher’s Note

All claims expressed in this article are solely those of the authors and do not necessarily represent those of their affiliated organizations, or those of the publisher, the editors and the reviewers. Any product that may be evaluated in this article, or claim that may be made by its manufacturer, is not guaranteed or endorsed by the publisher.

## Footnotes

1
https://www.who.int/news-room/fact-sheets/detail/breast-cancer
