# Supplementary material for: Repurposing Combination Therapy of Voacamine With Vincristine for Downregulation of Hypoxia-Inducible Factor-1α/Fatty Acid Synthase Co-axis and Prolyl Hydroxylase-2 Activation in ER+ Mammary Neoplasia
Source: Front Cell Dev Biol. 2021 Nov 18;9:736910. doi: 10.3389/fcell.2021.736910 (PMC8637442; doi:10.3389/fcell.2021.736910)
Supplement: Supplementary file 1 [file Data_Sheet_1.docx]

**Supplementary table2: List of compounds docked with PHD2**

| **Compound ID/Name** | **Compound Structure** | **Binding energy** | **Amino acids** | **Binding interaction** |
| --- | --- | --- | --- | --- |
| Vincristine |  | -10.46 | VAL401,  TYR403,  LYS402 | 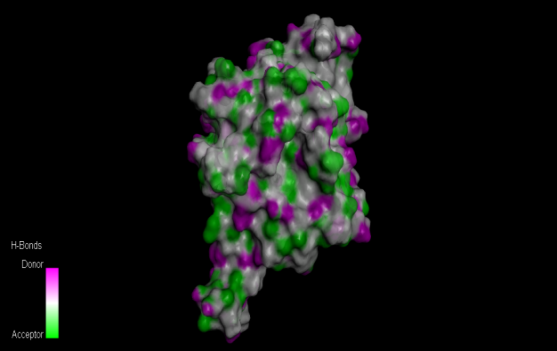 |
| Voacamine |  | -9.46 | LEU271,THR218LEU193,LEU191,HIS282,ARG282,ARG281,ALA190,LEU188,PHE213,GLY213,LEU214,GLU217,,ASP278,SER275, SER214, | 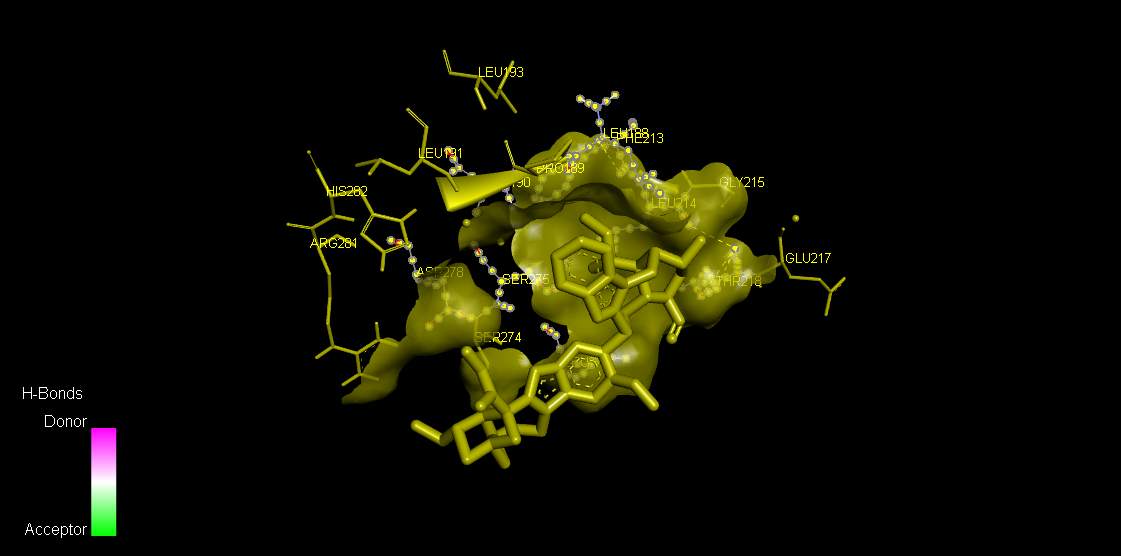 |
| Image-9915 |  | -13.04 | ARG322,TRP389,TYR310,ASP254GLN239,THR236,ILE256,TRP258,MET299,  TRP389 | 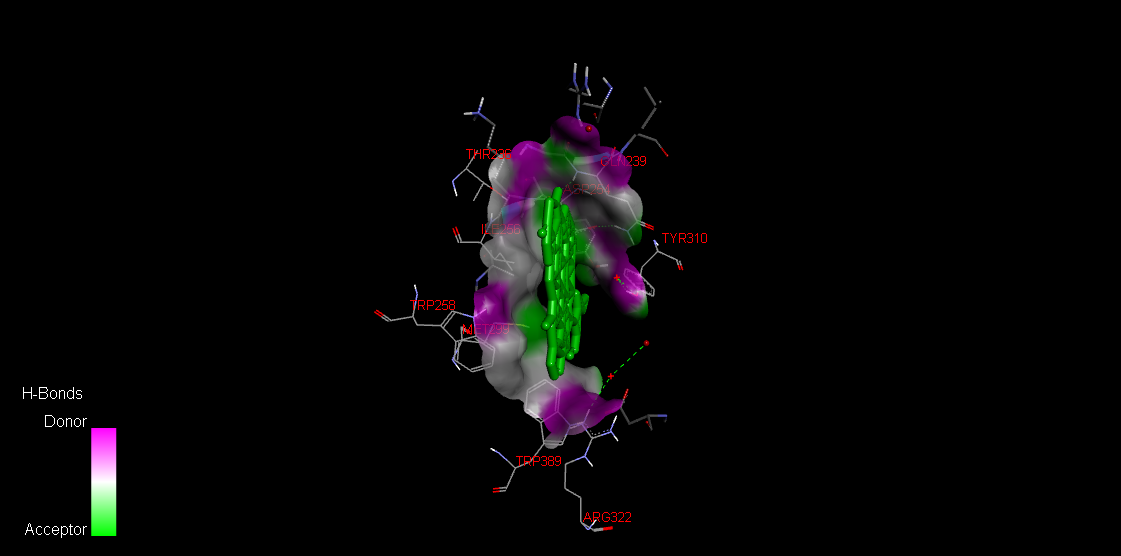 |
| Image-8266 |  | -10.00 | ALA336,  LYS337,  SER339,  GLY340,  ALA381,  TYR386 | 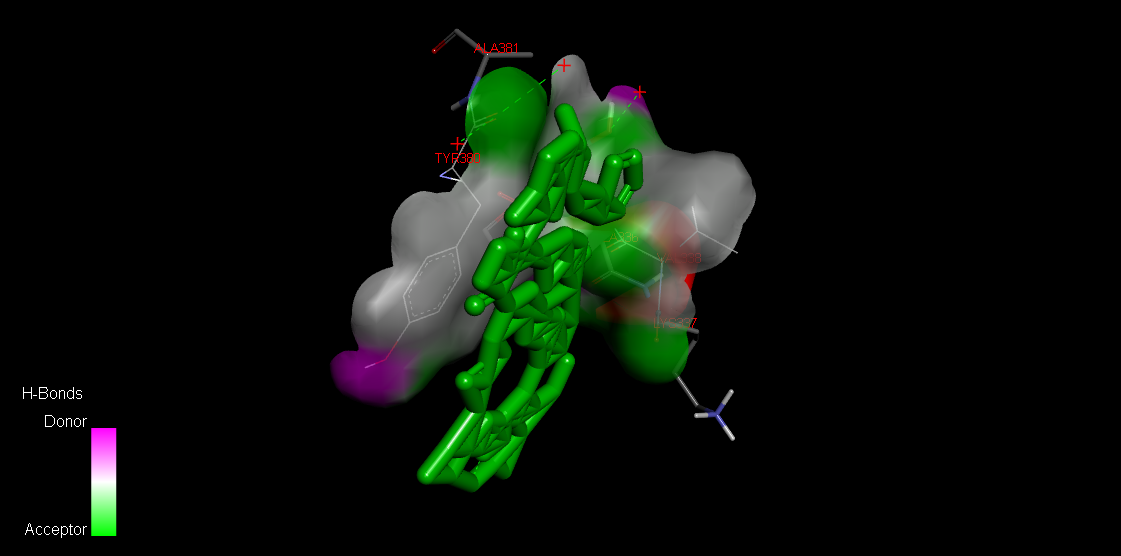 |
| Image-7112 |  | -8.46 | ALA381,  THR392,  LYS332,  ASP333 | 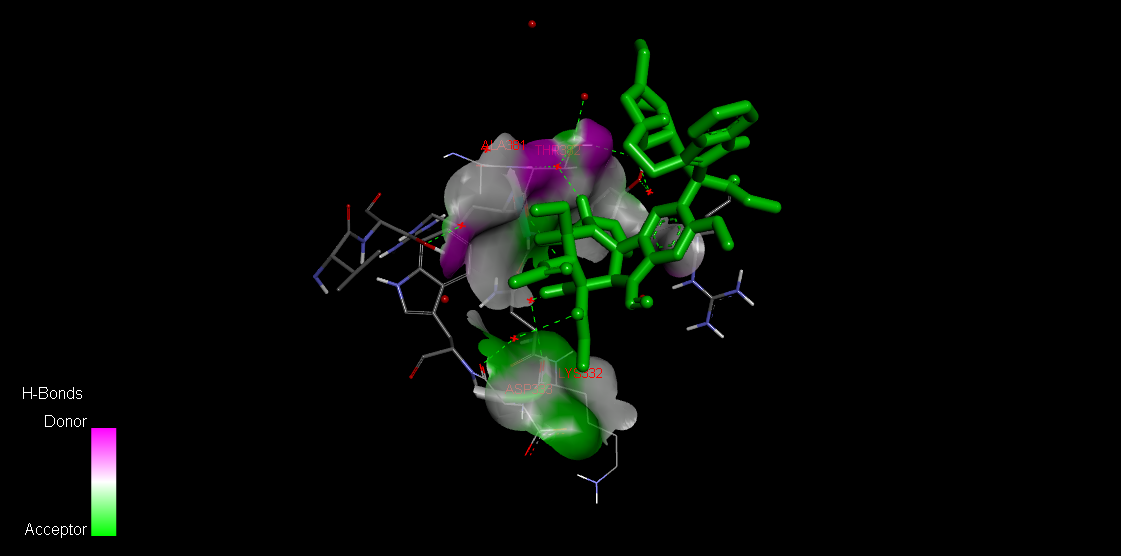 |
| Image-7078 |  | -7.52 | ASP224,  GLU225,ALA228,  THR232,  GLY265 | 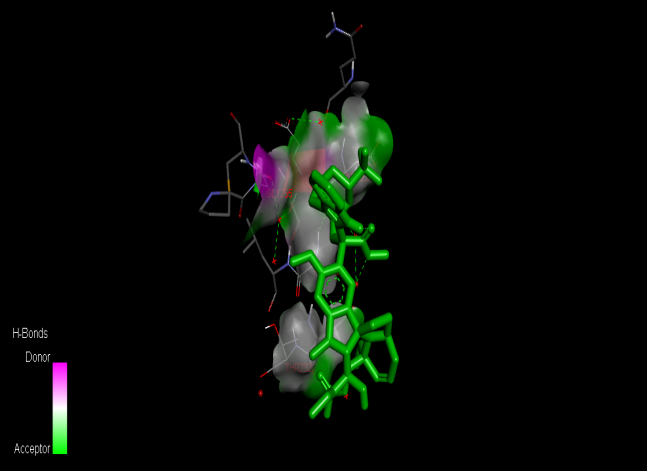 |
| Image-7036 |  | -8.12 | GLN239,ILE256,TRY258,  MET299,TRY389,TRY310,VAL311,ARG312,HIS313,VAL314,ASP315,  PRO317,ARG322,TRY389,ASN318 | 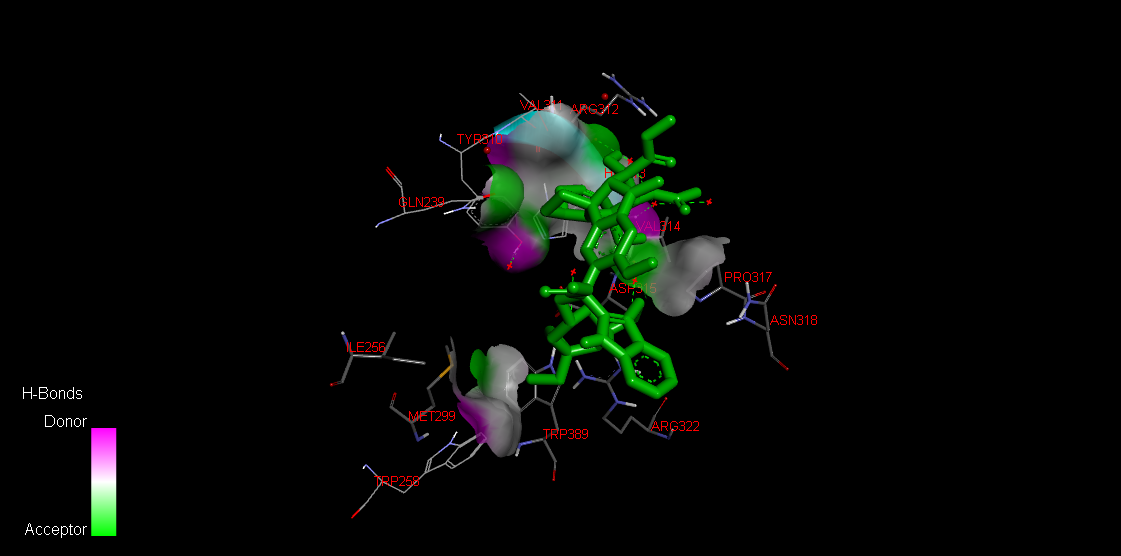 |
| Image-7025 |  | -7.07 | ASP237,PHE235,THR236,  ILE256,THR257,TRY258,  LYS297 | 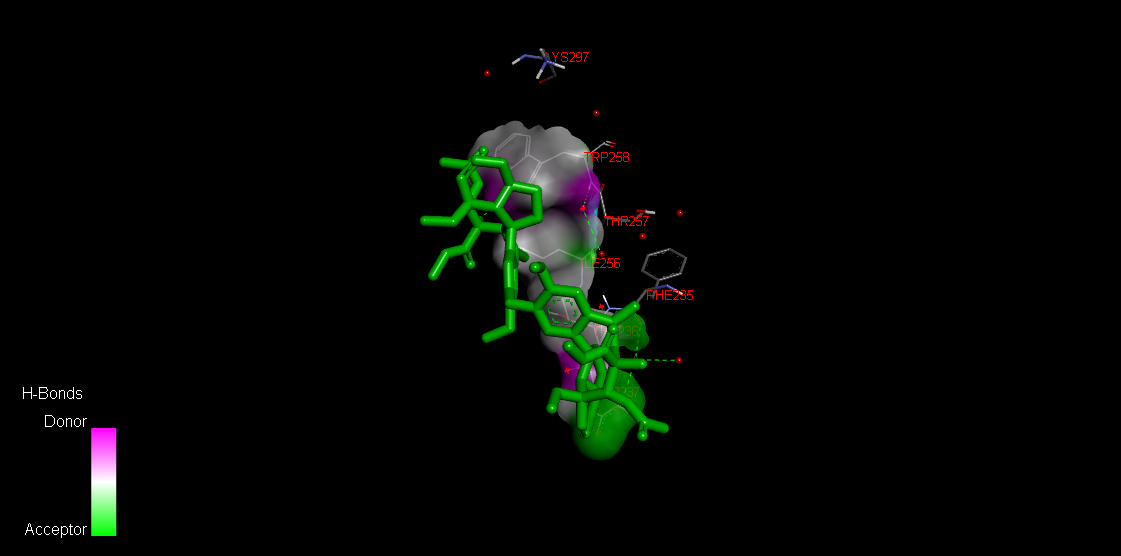 |
| Image-7004 |  | -8.93 | TRP258,  LYS297,  GLU263 | 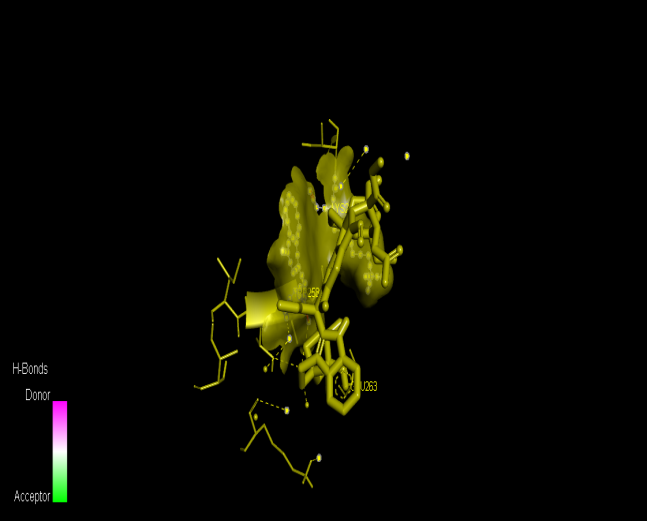 |
| Image-6958 |  |  | ARG398,GLU395,  GLY319,ASN318,  ASN316.PRO317,  ASP369, ARG370, TRP290, SER289ARG371, | 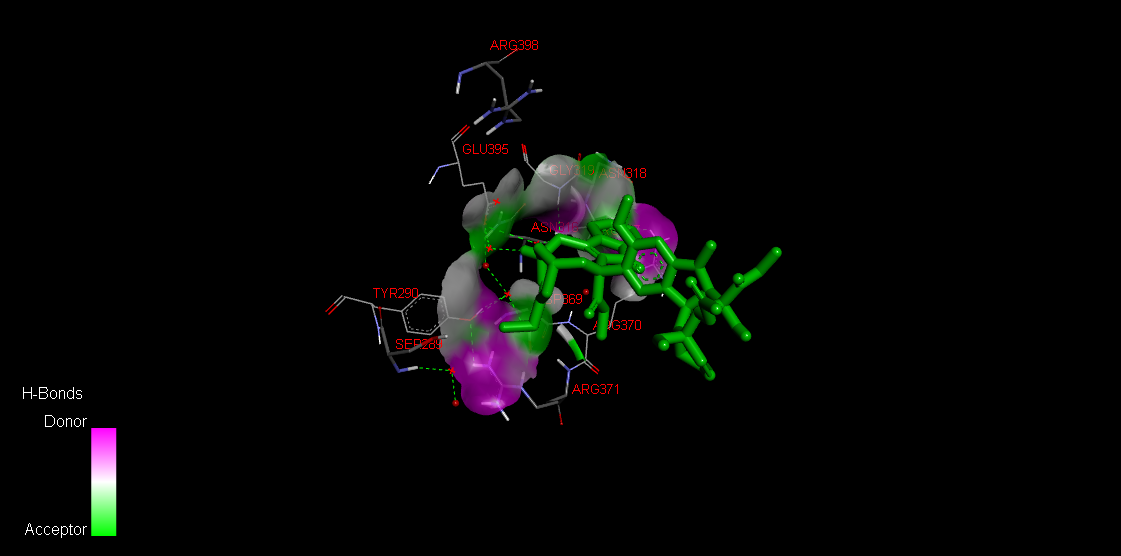 |
| Image-6401 |  | -7.37 | LYS297,  TRP258,  THR257,  THR236,  ILE259,  LYS234,  GLU263 | 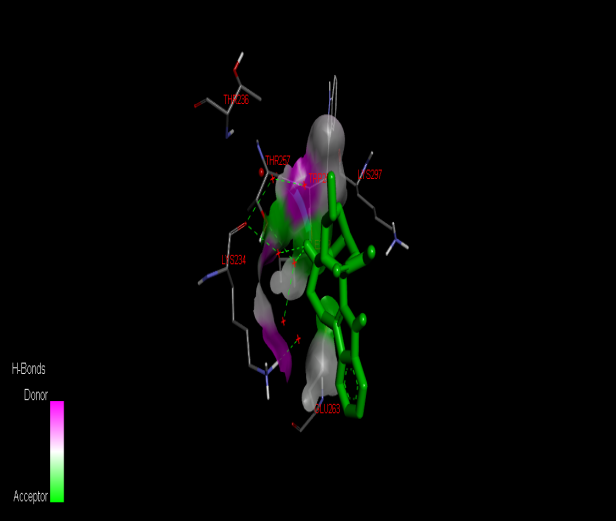 |
| Image-5914 |  | -6.12 | LYS234,  GLU263,  THR257,  TRP258,  LYS297 | 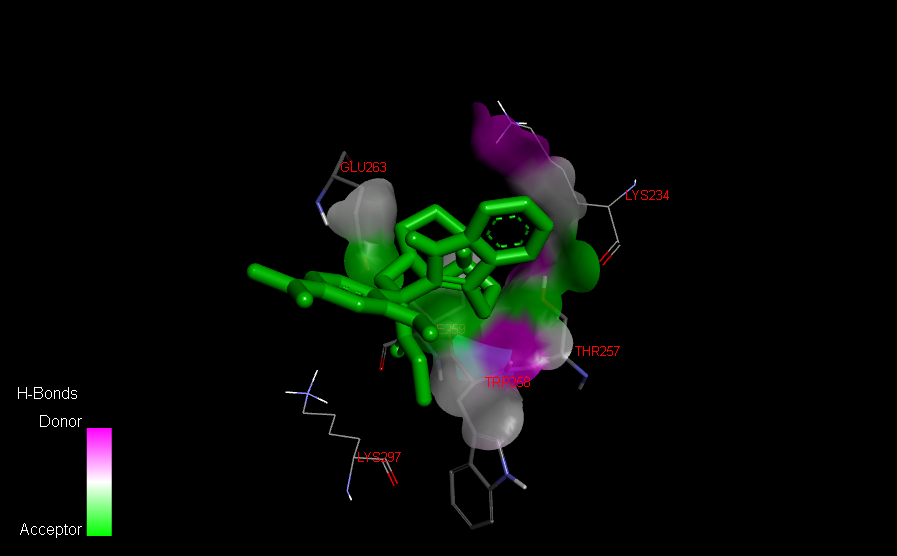 |
| Image-3255 |  | -10.60 | TRP258,  LYS297,  THR296 | 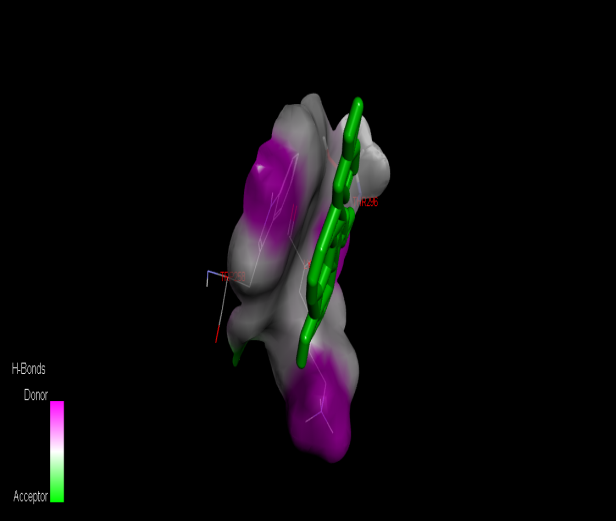 |
| Image-3241 |  | -9.06 | ASP333,  TRP334,  SER339 | 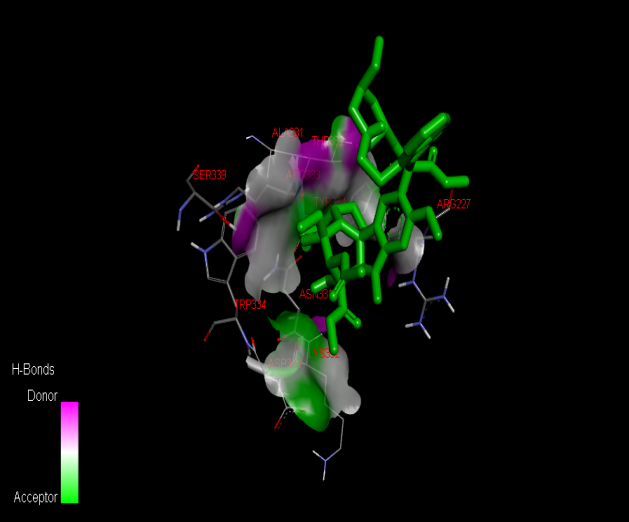 |
