# Supplementary material for: Repurposing Combination Therapy of Voacamine With Vincristine for Downregulation of Hypoxia-Inducible Factor-1α/Fatty Acid Synthase Co-axis and Prolyl Hydroxylase-2 Activation in ER+ Mammary Neoplasia
Source: Front Cell Dev Biol. 2021 Nov 18;9:736910. doi: 10.3389/fcell.2021.736910 (PMC8637442; doi:10.3389/fcell.2021.736910)
Supplement: Supplementary file 2 [file Table_1.docx]

**Supplementary table 1: Experimental design and treatment given**

| **S.No.** | **Groups** | **Treatment given** |
| --- | --- | --- |
| **1** | **Group1:Normal control (NC)** | **Saline 3ml/kg/p.o** |
| **2** | **Group2:Toxic control (TC)** | **DMBA (8mg/kg/i.v)** |
| **3** | **Group3:Treatment 1** | **VOA (1mg/kg/s.c.)** |
| **4** | **Group4:Treatment 2** | **VIN(1mg/kg)** |
| **5** | **Group5:Treatment 3** | **VOA (0.5mg/kg/s.c.)+VIN (0.5mg/kg/i.v.)** |
| **6** | **Group6:Treatment 4** | **VOA (1mg/kg/s.c.)+VIN (1mg/kg/i.v.)** |
| **7** | **Group7: DMSO control** | **DMSO 3%(2ml/kg/s.c)** |
